# Predictive and Prognostic Value of ^18^F-fluorodeoxyglucose Uptake Combined with Thymidylate Synthase Expression in Patients with Advanced Non-Small Cell Lung Cancer

**DOI:** 10.1038/s41598-019-48674-4

**Published:** 2019-08-21

**Authors:** Seung Hwan Moon, Jong-Mu Sun, Jin Seok Ahn, Keunchil Park, Byung-Tae Kim, Kyung-Han Lee, Myung-Ju Ahn, Joon Young Choi

**Affiliations:** 1 Department of Nuclear Medicine, Samsung Medical Center, Sungkyunkwan University School of Medicine, Seoul, Republic of Korea; 2 Division of Hematology-Oncology, Department of Medicine, Samsung Medical Center, Sungkyunkwan University School of Medicine, Seoul, Republic of Korea

**Keywords:** Cancer imaging, Non-small-cell lung cancer

## Abstract

We investigated the relationship between tumor ^18^F-fluorodeoxyglucose (FDG) uptake on positron emission tomography/computed tomography (PET/CT) scans and thymidylate synthase (TS) expression. In addition, we evaluated the value of FDG uptake in predicting treatment response and prognosis when combined with TS expression in patients with advanced non-small cell lung cancer (NSCLC). We measured the maximum standard uptake value, metabolic tumor volume, and total lesion glycolysis (TLG) of tumor lesions on pretreatment scan in 234 patients (age: 60.1 ± 9.4 years; males: 56.4%) with stage IV non-squamous NSCLC who were enrolled in the prospective phase II clinical trial. We investigated the correlation of the parameters with TS expression and the predictive values of the parameters compared with other clinical factors. Among these parameters, TLG was the most relevant parameter that had a significant correlation with TS expression (ρ = 0.192, *P* = 0.008). A multivariable Cox proportional-hazards model revealed that high TLG was a significant independent predictor for treatment response (hazard ratio [HR]: 2.05; *P* = 0.027), progression-free survival (HR: 1.39; *P* = 0.043), and overall survival (HR: 1.65; *P* = 0.035) with other factors. In patients with advanced non-squamous NSCLC, tumor TLG on pretreatment PET/CT scan has predictive and prognostic value.

## Introduction

Thymidylate synthase (TS) has a significant predictive and prognostic role in patients with non-squamous non-small cell lung cancer (NSCLC). TS plays an important role in DNA synthesis and folate metabolism; thus, the inhibition of this enzyme by chemotherapeutic agents such as pemetrexed induces the suppression of tumor growth^1–3^. As pemetrexed/cisplatin therapy has superior clinical benefits over other platinum-based doublet regimens^4,5^, and its chemotherapeutic outcomes are significantly associated with TS expression^6,7^, whether tumors express TS is an important issue in the management of non-squamous NSCLC. Given that obtaining multiple adequate specimens to represent the overall distribution of target protein expression levels in advanced cancer is difficult, finding an alternative biomarker for TS expression may be of interest.

The standardized uptake value (SUV) of a tumor measured by ^18^F-fluorodeoxyglucose (FDG) positron emission tomography/computed tomography (PET/CT) has the potential to be a valuable surrogate biomarker for TS^8^. FDG PET/CT is a noninvasive imaging method that enables the quantitation of tumor glucose metabolism and is now being widely used in oncology clinical practice^9,10^. This index has prognostic value as well as diagnostic usefulness in patients with NSCLC^11–15^. Moreover, it can clearly and easily image the metabolism within both primary and metastatic tumors, even if an adequate specimen is not obtained. A previous study demonstrated that TS expression was significantly correlated with glucose transporter 1 (Glut1) and hypoxia-inducible factor-1α (HIF-1α), key elements for determining the amount of FDG uptake within tumor cells, in patients with primary lung cancer^16^. There have been few attempts to evaluate the association between tumor FDG uptake and TS expression^8^. Kaira *et al*. recently investigated the relationship between these two factors in patients with various thoracic neoplasms and reported a significant correlation in lung adenocarcinoma^8^. However, the study was limited by a retrospective study design, a small number of subjects with resectable lung cancer, and a lack of comparisons with traditional risk factors. The precise relationship of tumor FDG uptake with TS expression therefore remains to be explored. Furthermore, with respect to TS expression and TS inhibitor treatment, the role of tumor FDG uptake in prediction and prognosis also remains unclear.

We therefore investigated the relationship between tumor FDG uptake and TS expression in the prospectively collected dataset for our previous stratified randomized phase II clinical trial^4^ and assessed the value of tumor FDG uptake in predicting treatment response to TS inhibitors and the prognosis of the patients who were stratified by TS expression level.

## Materials and Methods

### Patients

Study candidates consisted of 315 patients with histologically confirmed advanced non-squamous NSCLC, not including large-cell neuroendocrine carcinoma, who were enrolled in a previous prospective phase II clinical trial (NCT01401192) to investigate whether TS expression is a predictive marker for the clinical outcome of pemetrexed/cisplatin chemotherapy^4^. All candidates were aged 18 years or older, had no previous history of systemic therapy for advanced NSCLC, had an adequate Eastern Cooperative Oncology Group performance status (0 or 1), had adequate renal function (estimated creatinine clearance ≥ 50 mL/min), had one or more measurable lesions per Response Evaluation Criteria in Solid Tumors, version 1.1 (RECIST 1.1), and received at least one dose of study chemotherapy. Exclusion criteria included uncontrolled diabetes mellitus, heart disease, obstructive pneumonia, infection, and uncontrolled symptomatic brain metastasis. From July 2011 to January 2014, the subjects provided written informed consent and participated in the trial conducted at our institution.

Sixty-one of these patients were excluded because of a lack of pretreatment PET/CT (n = 47), PET/CT data loss (n = 1), or a substantial interval (60 days) between pretreatment PET/CT scan and chemotherapy (n = 13). Additionally, 3 patients who had pleomorphic carcinoma (n = 1) or carcinoma not otherwise specified (n = 2) and 17 patients who had recurrent (n = 12) or stage III (n = 5) disease were excluded. Thus, from this prospectively collected dataset, a total of 234 patients with stage IV adenocarcinoma who underwent PET/CT prior to chemotherapy were ultimately included for analysis. This study was approved by our Institutional Review Board, and the requirement for written informed consent was waived.

### Treatment and follow-up

The treatment regimen and follow-up protocol were previously described in detail^4^. Briefly, patients were stratified into TS-negative or TS-positive groups. TS-positive was defined as TS expression in more than 10% of tumor cells. After stratification, patients were randomly assigned to receive either gemcitabine/cisplatin or pemetrexed/ cisplatin therapy for a maximum of six cycles until disease progression or an unacceptable adverse event. Maintenance chemotherapy was not allowed after the completion of the planned chemotherapy. The response was assessed by CT scans according to RECIST 1.1. CT scans were performed every two cycles of the planned treatment. After completion of the treatment, CT scans were repeated every 2 months. Among these serial evaluations, the best response was taken for the response rate evaluation. Complete or partial responses were regarded as objective responses, and the percentage of these responses among all treated patients, including patients who were not evaluable, was calculated as the objective response rate. If clinically indicated, other diagnostic work-ups were performed at the physician’s discretion. Radiologists were blinded to treatment allocation information and performed an independent review.

Overall survival (OS) was defined as the time from the date of pretreatment PET/CT scan to death as a result of any cause, with censoring at the date of the final follow-up in surviving patients. Progression-free survival (PFS) was defined as the time from the date of pretreatment PET/CT scan to disease progression, with censoring at the date of the final follow-up if the patient had not progressed.

### PET/CT imaging

All subjects fasted for at least 6 h before the PET/CT study, and blood glucose levels were <200 mg/dl at the time of FDG injection. Imaging was performed in the majority of subjects (n = 191, 81.6%) on a GE STE PET/CT scanner (Milwaukee, WI, USA), while a GE Discovery LS PET/CT scanner was used for the other subjects. At 60 min after the injection of 225–417 MBq FDG, transmission scans were acquired at 4 min per frame in 2D mode (Discovery LS) or 2.5 min per frame in 3D mode (Discovery STe). Whole-body spiral CT was performed with an 8-slice helical CT (140 KeV, 40 to 120 mAs adjusted to body weight; section width = 5 mm) for the Discovery LS scanner and with a 16-slice helical CT (140 KeV, 30 to 170 mAs with AutomA mode; section width = 3.75 mm) for the STe scanner. Attenuation-corrected PET images (voxel size = 4.3 × 4.3 × 3.9 mm for Discovery LS, 3.9 × 3.9 × 3.3 mm for Discovery STe) were reconstructed using CT data and 2D (28 subsets, 2 iterations; Discovery LS) or 3D ordered-subset expectation maximization algorithms (20 subsets, 2 iterations; STe). The median time interval between pretreatment PET/CT and the initiation of chemotherapy was 10 days (range, 0–51 days). The median time interval between PET/CT scan and biopsy was 14 days (range, 0–50 days).

### PET/CT image analysis

Two experienced nuclear medicine physicians reviewed all PET/CT images and measured the tumor maximum standard uptake value (SUV_max_), metabolic tumor volume (MTV), and total lesion glycolysis (TLG) for analysis using MIM version 6.4 software (MIM Software Inc., Cleveland, OH, USA). To investigate the correlation between tumor FDG uptake and TS protein expression, SUV_max_, MTV, and TLG were measured in the lesion in which the biopsy was performed. More specifically, 180 patients (180/234; 76.9%) had biopsy sites consistent with the primary tumor, and the remaining 54 patients (54/234; 23.1%) had metastatic lesions consisting of 17 bone, 12 extrathoracic lymph node, 19 mediastinal lymph node, 2 brain, and 4 other lesions (adrenal gland, kidney, muscle, and trachea). The target tumors were identified by experienced nuclear medicine physicians who were unaware of clinical information except for the target tumor site. As the physician drags the cursor out from the center of the target tumor to a point near the edge of the lesion, the software automatically outlines a three-dimensional volume of interest (VOI) on the tumor. After creating a gradient-based segmentation, we created intensity-based segments by changing thresholds based on mediastinal blood pool (MBP) activity, liver activity, and absolute SUV. For thresholds using MBP and liver activities, a VOI consisting of 5 × 5 × 1 voxels was manually drawn at the aortic arch and right hepatic lobe at the level of the hepatic hilum, and the average SUV plus two standard deviations of each VOI was adopted as the threshold. Absolute SUV thresholds were fixed values of SUV 2.5. Therefore, 3 different segmentations were automatically generated, and 7 different PET parameters were obtained.

### Statistical analysis

The correlations of different continuous covariates including SUV_max_, MTV, TLG, and TS expression were assessed using Spearman’s rank test. Receiver operating characteristic (ROC) curve analysis was used to determine the most appropriate cut-off values of the PET parameters in predicting TS expression.

For continuous variables, we used the independent-samples t-test to compare means between groups, and we used the chi-square test to determine whether there was a significant difference between groups for categorical variables. Objective response rates according to tumor metabolic activity in combination with other categorical covariates including TS expression and treatment arms were compared.

Logistic regression analysis and Cox regression models were used to identify predictors for treatment response and for prognosis, respectively. In each univariate analysis, all covariates including age, sex, smoking status, epidermal growth factor receptor (EGFR) mutation, ALK rearrangement, TS expression positivity, treatment arm, and tumor metabolic activity were included. After that, significant variables from the univariate analyses were considered for multivariate analysis, and a final model was obtained using the enter method with α = 0.05 for insertion and α = 0.1 for deletion of each covariate. The PFS and OS according to the variables were estimated using the Kaplan-Meier method and compared using the log-rank test.

SPSS for Windows (version 16.0, SPSS Inc., Chicago, IL, USA) was used and two-sided *P*-values of <0.05 were considered significant.

## Results

### Patient characteristics

**Figure 1:**
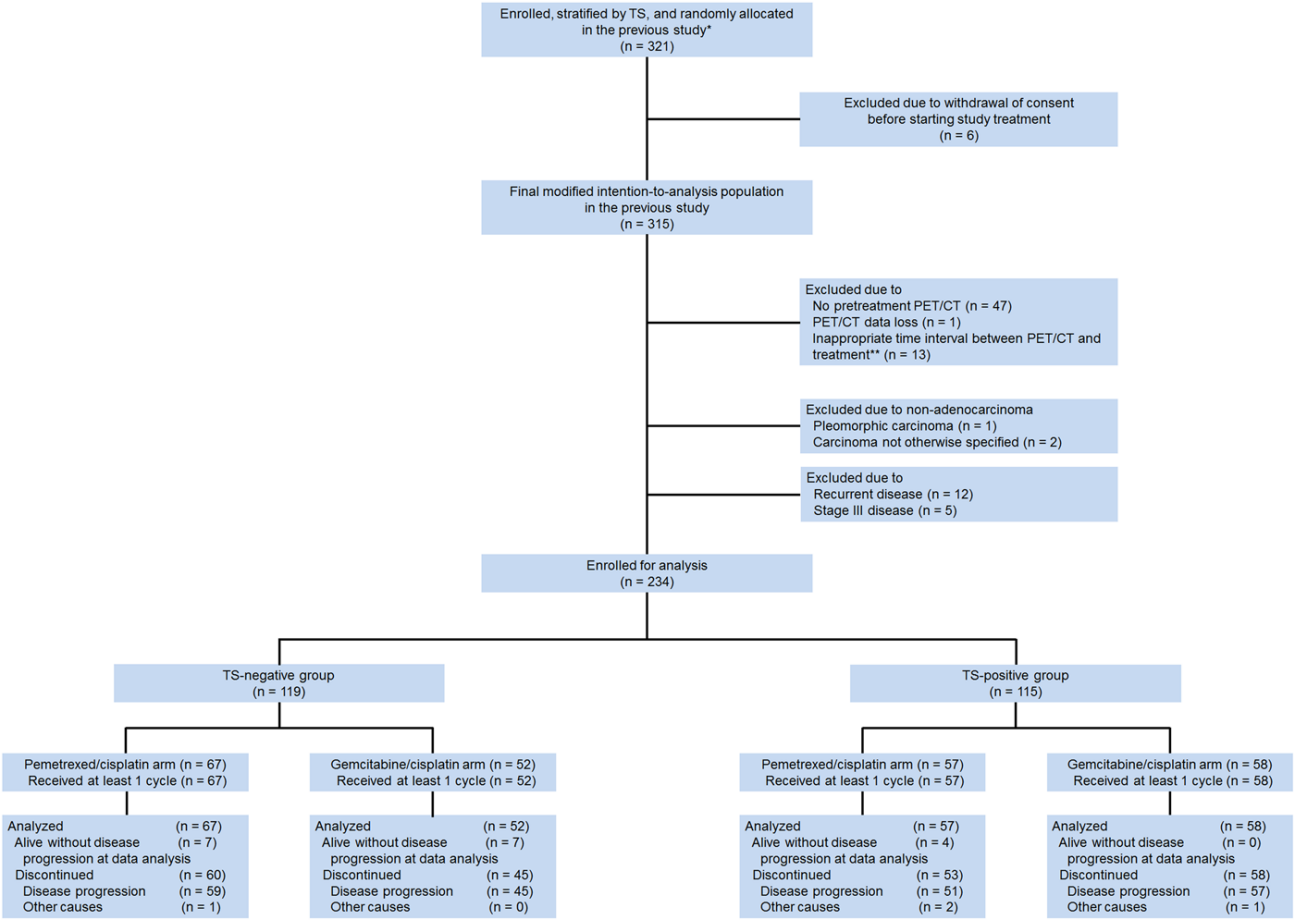
Flow diagram of the study population. *Prospective phase II clinical trial (NCT01401192); PET/CT, positron emission tomography/computed tomography; **, ≥60 days; TS, thymidylate synthase; other causes including death due to adverse event, disease progression, or unknown cause.

In total, 234 patients stratified by TS expression status as either TS-negative or TS-positive were enrolled in the study (Fig. 1). The clinical characteristics of the study patients according to the TS expression are summarized in Table 1. There were significant differences between the TS-positive group and the TS-negative group in sex (*P* = 0.016), smoking (*P* = 0.004), EGFR mutation (*P* = 0.015), TLG (*P* = 0.048), death (*P* = 0.001), and OS (*P* = 0.001).

**Table 1:** Patient Characteristics (N = 234).

Characteristic		Total	TS expression		
		N (%)	Positive (N = 115)	Negative (N = 119)	*P*-value
Age	≥65 years	80 (34.2%)	45 (39.1%)	35 (29.4%)	0.118
Male sex		132 (56.4%)	74 (64.3%)	58 (48.7%)	0.016
Smoking					0.004
	Never smoker	106 (45.3%)	42 (36.5%)	64 (53.8%)	
	Former smoker	78 (33.3%)	39 (33.9%)	39 (32.8%)	
	Current smoker	50 (21.4%)	34 (29.6%)	16 (13.4%)	
EGFR mutation					0.015
	Positive	77 (32.9%)	28 (24.3%)	49 (41.2%)	
	Negative	143 (61.1%)	81 (70.4%)	62 (52.1%)	
	Unknown	14 (06.0%)	6 (5.2%)	8 (6.7%)	
ALK rearrangement					0.822
	Positive	14 (06.0%)	4 (3.5%)	10 (8.4%)	
	Negative	219 (93.6%)	110 (95.7%)	109 (91.6%)	
	Unknown	1 (00.4%)	1 (0.9%)	0 (0.0%)	
Treatment					0.303
	Pemetrexed/cisplatin	124 (53.0%)	57 (49.6%)	67 (56.3%)	
	Gemcitabine/cisplatin	110 (47.0%)	58 (50.4%)	52 (43.7%)	
SUV_max_	Mean ± SD	10.2 ± 4.8	10.7 ± 5.0	9.6 ± 4.5	0.086
MTV	Mean ± SD	58.5 ± 92.2	64.7 ± 97.7	44.7 ± 69.8	0.074
TLG	Mean ± SD	309.7 ± 539.9	351.9 ± 583.5	225.1 ± 371.9	0.048
Progression		212 (90.6%)	108 (93.9%)	104 (87.4%)	0.080
Death		104 (44.4%)	64 (55.7%)	40 (33.6%)	0.001
PFS	Median, IQR (months) 5.6, 3.2–7.4		5.5, 3.3–6.9	6.1, 3.0–8.9	0.060
OS	Median, IQR (months) 13.7, 7.5–21.3		11.4, 6.7–18.2	15.8, 10.0–25.6	0.001

EGFR, epidermal growth factor receptor; ALK, anaplastic lymphoma kinase; TS, thymidylate synthase; SUV_max_, maximum standard uptake value; SD, standard deviation; MTV, metabolic tumor volume; TLG, total lesion glycolysis; PFS, progression-free survival; IQR, interquartile range; OS, overall survival.

### Tumor FDG uptake and TS expression

The PET parameters of the tumor had very weak positive correlations with TS expression (Supplementary Table 1). Among them, TLG_Li_ was revealed to be the most relevant one in this study (Spearman’s correlation coefficient = 0.192, *P* = 0.008). The appropriate cut-off value for TLG_Li_ in predicting the TS positivity was determined by ROC analysis. As a result, a TLG_Li_ value greater than 88.7 was selected as a criterion, and tumor FDG uptake was classified into two groups according to the measured TLG_Li_; tumors with TLG_Li_ values greater than 88.7 were regarded as having high TLG, and others were regarded as having low TLG. The categorized tumor metabolic activities were included for further analysis.

Despite the unstable relationship, the positive rate of TS expression in patients with high TLG was significantly higher than that in patients with low TLG (60.8% vs. 41.1%, *P* = 0.007; Supplementary Table 3). In addition, a significant difference in TS expression was also observed according to EGFR mutation. Patients with wild-type EGFR had a higher rate of positive TS expression than did patients with EGFR mutations (55.4% vs. 36.4%, *P* = 0.014; Supplementary Table 3). However, no significant difference in TS expression according to the presence of anaplastic lymphoma kinase (ALK) gene rearrangement was noted.

### Predictive significance of tumor FDG uptake

#### Response according to TS expression status

**Figure 2:**
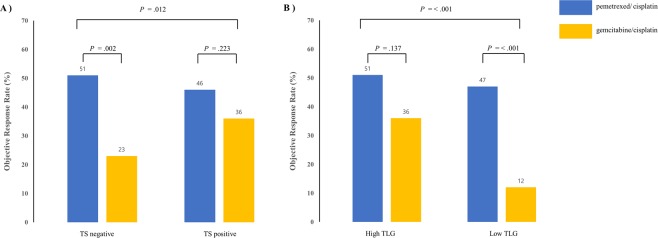
Response rates according to biomarkers and treatment arm. (**A**) The frequency of objective response from patients with pemetrexed/cisplatin was prominent compared to that of the patients treated with gemcitabine/cisplatin in the TS-negative group. (**B**) The response rate for the pemetrexed/cisplatin arm was significantly higher than that for the gemcitabine/cisplatin arm in the low TLG group, whereas response rates were not significantly different between the treatment arms in the high TLG group. TS, thymidylate synthase

Response to treatment differed according to TS expression status in this dataset (*P* = 0.012; Fig. 2A). Patients treated with pemetrexed/cisplatin had a higher response rate than those treated with gemcitabine/cisplatin in the TS-negative group, but not in the TS-positive group (Fig. 2A).

#### Response according to TLG

The treatment response differed significantly according to tumor FDG uptake. Patients with high TLG had higher response rates than did patients with low TLG (*P* = 0.038). More specifically, patients treated with gemcitabine/cisplatin showed significantly lower response rates when they had low TLG (Fig. 2B). The response rates were 51% for the pemetrexed/cisplatin treatment and 36% for the gemcitabine/cisplatin treatment (*P* = 0.137) in the high TLG group, while the response rates were 47% for the pemetrexed/cisplatin treatment and 12% for the gemcitabine/cisplatin treatment (*P* < 0.001) in the low TLG group (Fig. 2B).

#### Response according to TLG and TS expression status

**Figure 3:**
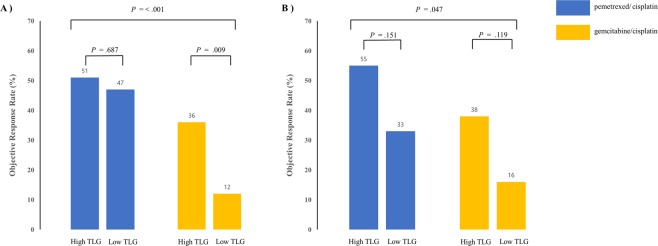
Treatment response according to TLG in (**A**) the TS-negative group and (**B**) the TS-positive group. TS, thymidylate synthase.

Interestingly, response rates according to TLG were further stratified by TS expression status (Fig. 3). The difference in response rates to the gemcitabine/cisplatin treatment between the high TLG group and the low TLG group was significant in the TS-negative group (Fig. 3A, 36% vs. 12%, *P* = 0.009), but not in the TS-positive group (Fig. 3B, 38% vs. 16%, *P* = 0.119). On the other hand, the difference in response rates to the pemetrexed/cisplatin treatment according to tumor TLG seemed to be more prominent in the TS-positive group than in the TS-negative group. However, it was not statistically significant (Fig. 3B, *P* = 0.151).

#### Results of logistic regression analysis

Upon univariate analysis to identify the parameters predicting treatment response, high TLG of tumor, male sex, and pemetrexed/cisplatin treatment were significantly associated with objective response (Table 2). Positive TS expression, EGFR mutation, smoking, and old age were categorical variables that failed to show a significant association with treatment response. A multivariate analysis that included significant univariate factors revealed that the high TLG (vs. low TLG; hazard ratio [HR]: 2.05; 95% CI: 1.08–3.88; *P* = 0.027), pemetrexed/cisplatin treatment (HR: 3.15; 95% CI: 1.64–6.04; *P* = 0.001), and male sex (HR: 0.44; 95% CI: 0.23–0.83; *P* = 0.011) were significant independent predictors for response (Table 2).

**Table 2:** Factors Associated with Objective Response Rate.

	Univariate			Multivariate		
	OR	95% CI	*P*-value	OR	95% CI	*P*-value
AP group (vs. GP group)	2.28	1.33–3.93	0.003	3.15	1.64–6.04	0.001
High TLG (vs. low TLG)	1.88	1.03–3.43	0.038	2.05	1.08–3.88	0.027
Male (vs. female)	0.56	0.33–0.96	0.034	0.44	0.23–0.83	0.011
EGFR wild-type (vs. mutation)	1.20	0.69–2.12	0.517			
TS-positive (vs. TS-negative)	1.06	0.63–1.79	0.833			
Ever smoked (vs. never smoker)	0.91	0.54–1.54	0.722			
Age ≥65 years (vs. <65 years)	0.68	0.51–1.55	0.890			
ALK rearrangement	1.18	0.39–3.53	0.764			

OR, odds ratio; CI, confidence interval; AP, pemetrexed/cisplatin-treated; GP, gemcitabine/cisplatin-treated; EGFR, epidermal growth factor receptor; TS, thymidylate synthase; ALK, anaplastic lymphoma kinase.

### Prognostic significance of tumor FDG uptake

**Figure 4:**
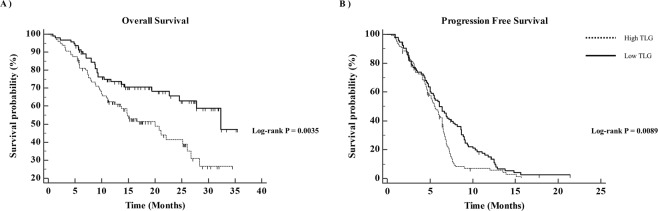
**(A**) Overall survival and (**B**) progression-free survival according to tumor FDG uptake. FDG, ^18^F-fluorodeoxyglucose.

**Figure 5:**
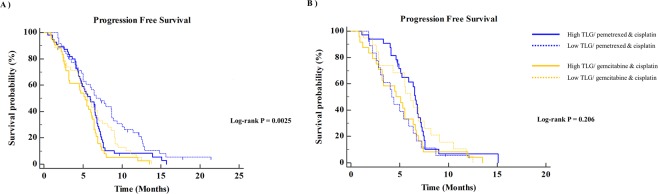
Progression-free survival of the pemetrexed/cisplatin and gemcitabine/cisplatin arms stratified by tumor FDG uptake in (**A**) the TS-negative group and (**B**) the TS-positive group. TS, thymidylate synthase; FDG, ^18^F-fluorodeoxyglucose.

**Figure 6:**
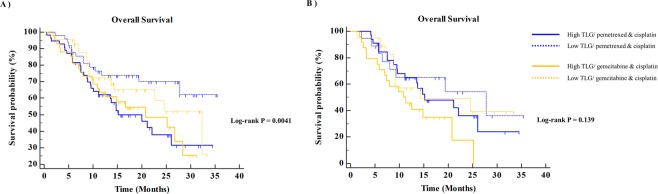
Overall survival of the pemetrexed/cisplatin and gemcitabine/cisplatin arms stratified by tumor FDG uptake in (**A**) the TS-negative group and (**B**) the TS-positive group. TS, thymidylate synthase; FDG, ^18^F-fluorodeoxyglucose.

The prognoses of patients differed significantly according to tumor FDG uptake. Patients with high TLG showed poor OS and poor PFS compared to patients with low TLG (*P* = 0.0035 and *P* = 0.0089, respectively; Fig. 4). The survival of patients stratified tumor FDG uptake, and the treatment was subdivided by TS expression status (Figs 5 and 6). In patients with TS-negative tumors, PFS and OS were significantly different according to the tumor TLG and the treatment (*P* = 0.037 and *P* = 0.041, respectively; Figs 5A and 6A). However, in patients with TS-positive tumors, there were no significant differences in PFS and OS according to the tumor TLG or the treatment (*P* = 0.206 or *P* = 0.139, respectively; Figs 5B and 6B).

Upon multivariate analysis for OS, EGFR wild type (HR: 2.00; 95% CI: 1.15–3.48; *P* = 0.014), positive TS expression (HR: 1.69; 95% CI: 1.05–2.72; *P* = 0.030), elderly age (HR: 1.94; 95% CI: 1.23–3.07; *P* = 0.004), and high TLG (HR: 1.65; 95% CI: 1.04–2.63; *P* = 0.035) were significant predictors for poor OS (Table 3). For PFS, high TLG (HR: 1.39; 95% CI: 1.01–1.93; *P* = 0.043) and pemetrexed/cisplatin (HR: 0.68; 95% CI: 0.49–0.93; *P* = 0.017) showed significant association with disease progression (Table 4).

**Table 3:** Factors Associated with Poor Overall Survival.

	Univariate			Multivariate		
	HR	95% CI	*P*-value	HR	95% CI	*P*-value
Male (vs. female)	2.14	1.41–3.27	0.004	1.22	0.57–2.62	0.602
EGFR wild-type (vs. mutation)	2.69	1.65–4.40	<0.001	2.00	1.15–3.48	0.014
TS-positive (vs. TS-negative)	2.11	1.42–3.15	<0.001	1.69	1.05–2.72	0.030
Ever smoked (vs. never smoker)	2.18	1.44–3.31	<0.001	1.61	0.79–3.41	0.214
Age ≥65 years (vs. <65 years)	1.99	1.35–2.94	<0.001	1.94	1.23–3.07	0.004
High TLG	1.94	1.23–3.06	0.004	1.65	1.04–2.63	0.035
AP group (vs. GP group)	1.01	0.68–1.48	0.974			
ALK rearrangement	0.62	0.23–1.68	0.345			

HR, hazard ratio; CI, confidence interval; EGFR, epidermal growth factor receptor; TS, thymidylate synthase; TLG, total lesion glycolysis; AP, pemetrexed/cisplatin-treated; GP, gemcitabine/cisplatin-treated; ALK, anaplastic lymphoma kinase.

**Table 4:** Factors Associated with Poor Progression-Free Survival.

	Univariate			Multivariate		
	HR	95% CI	*P*-value	HR	95% CI	*P*-value
Male (vs. female)	1.53	1.16–2.01	0.003	1.51	0.95–2.39	0.080
Ever smoked (vs. never smoker)	1.35	1.03–1.77	0.030	0.92	0.57–1.46	0.708
High TLG	1.50	1.10–2.03	0.010	1.39	1.01–1.93	0.043
Age ≥65 years (vs. <65 years)	1.35	1.02–1.80	0.037	1.27	0.91–1.78	0.175
TS-positive (vs. TS-negative)	1.49	1.12–1.96	0.005	1.25	0.90–1.73	0.181
AP group (vs. GP group)	0.71	0.54–0.94	0.015	0.68	0.49–0.93	0.017
EGFR wild-type (vs. mutation)	1.24	0.93–1.64	0.148			
ALK rearrangement	0.83	0.47–1.45	0.504			

HR, hazard ratio; CI, confidence interval; TLG, total lesion glycolysis; TS, thymidylate synthase; AP, pemetrexed/cisplatin-treated; GP, gemcitabine/cisplatin-treated; EGFR, epidermal growth factor receptor; ALK, anaplastic lymphoma kinase.

### Subgroup analyses

We additionally performed subgroup analyses to investigate the effects of using two PET/CT scanners with different reconstruction methods on the results. The major findings were not changed in the subgroup analyses with the majority of the enrolled patients who underwent PET/CT scan on GE STE scanner (n = 191, 81.6%). TLG has a weak correlation with TS expression (correlation coefficient = 0.2394, *P* = 0.0027, Supplementary Table 2) and high TLG showed association with the positive rate of TS expression (Odds ratio = 2.64, *P = *0.003, Supplementary Table 4). High TLG was a significant independent predictor for response (HR: 2.31; 95% CI: 1.12–4.73; *P* = 0.023), poor OS (HR: 1.74; 95% CI: 1.01–3.01; *P* = 0.046), and poor PFS (HR: 1.49; 95% CI: 1.06–2.09; *P* = 0.020) on multivariable analyses (Supplementary Tables 5, 6 and 7, respectively). In addition, the pattern of treatment response according to TLG and treatment arm was similar to the result of the whole group (Supplementary Figure).

## Discussion

In this study, TLG of tumor on pretreatment PET/CT scan was an independent predictor of TS expression, treatment response, and clinical outcome.

As the evidence indicating an association between TS expression and clinical outcomes in patients who received pemetrexed-based therapy has grown^4,6,7^, there have been attempts to identify a biomarker related to TS expression^8,16^. Results from previous studies demonstrated that the TS expression level is associated with tumor FDG uptake^8,16–18^. Kaira *et al*. reported that TS expression has a significant correlation with Glut1, HIF-1α^16^, and FDG uptake^8^. These findings correspond to the well-known biological regulatory mechanism, where Glut1 determines FDG uptake within tumor cells and HIF-1α regulates Glut1 expression^17^.

In this study, correlations between PET parameters and TS expression level were significant, and the positive rate of TS expression was also significantly higher in patients with high TLG than in patients with low TLG. Nevertheless, the relationship was weak (*r* = 0.192, *P* = 0.008). This result was not consistent with the previous study conducted by Karia *et al*.^8^; they demonstrated that tumor FDG uptake had a moderate positive relationship with TS expression in patients with adenocarcinoma (*r* = 0.596, *P* < 0.001). The inconsistent results may be due to the different characteristics of the study subjects. We enrolled 234 patients with stage IV adenocarcinoma, whereas they included 95 patients with adenocarcinoma who underwent surgical management, which means they had relatively early stages of lung cancer. In addition, the median value of measured tumor SUV_max_ in our study was higher than that of the previous study (9.3 vs. 5.2). Given that the FDG uptake of a tumor might reflect the tumor aggressiveness^19–21^, our study population probably had more advanced and aggressive tumors than their counterparts in the prior study, which may have affected the results.

In the previous study involving this dataset, TS expression was predictive of treatment response according to the treatment regimen^4^. Compared with gemcitabine/cisplatin, the response rate of pemetrexed/cisplatin was prominent in the TS-negative group rather than in the TS-positive group. In this study, TLG was predictive of treatment response. Interestingly, TLG seems to stratify the response rate further for the gemcitabine/cisplatin treatment in the TS-negative group. In other words, the predictive value of TLG appears to be more prominent in patients with TS-negative tumors who underwent gemcitabine/cisplatin treatment. In addition, with regard to PFS and OS, tumor TLG was an independent prognostic marker. TLG was able to stratify the survival curve divided by the treatment arm and it differed according to the TS expression status. Our results are quite unique and provide several unexpected findings.

First, although there have been many studies on the prognostic value of tumor FDG uptake in lung cancer^9–11^, little is known about the value of this biomarker in predicting objective treatment response to certain chemotherapy regimens. High TLG as a predictor of good treatment response seems contradictory to the results of previous studies, because it has been reported that high tumor FDG uptake is closely related to tumor aggressiveness^19–21^ and thus is associated with poor clinical outcomes in lung cancer^9,10^. Multivariable analyses in this study also demonstrated that high TLG is predictive of poor OS. A close look at the clinical course of the study patients and at the cancer biology provides a convincing explanation. Patients with high tumor FDG uptake were more likely to respond to treatment, but they were also more likely to experience disease progression. On the other hand, patients with low tumor FDG uptake were less likely to respond to treatment, but they were also less likely to have disease progression. As a result, patients with low tumor FDG uptake may show the best prognosis if they respond to treatment.

Second, given that FDG uptake reflects the proliferation activity of the tumor^19–21^, a higher response rate following pemetrexed/cisplatin therapy in patients with high tumor FDG uptake than in patients with low FDG uptake is a persuasive result, because pemetrexed is classified as an antimetabolite, which may be more effective against aggressive tumor cells that grow quickly^22^. This assumption could also explain why the gemcitabine/cisplatin-treated group had a significant difference in treatment response according to tumor FDG uptake. Gemcitabine is a nucleoside analog that inhibits DNA synthesis and enzymes related to deoxynucleotide metabolism, and is classified as an antimetabolite^23^. According to the above-mentioned hypothesis, patients with high tumor FDG uptake in the gemcitabine/cisplatin-treated group have a higher response rate than do patients with low FDG uptake.

The present study has several limitations. Although this study used a prospectively collected dataset, the retrospective analysis limits the validity of the study findings. In addition, almost 26% of the candidates were excluded due to several reasons described in the methods. The large number of dropouts may weaken the statistical power for robust analysis. The methodological issue regarding the use of two PET/CT scanners with different reconstruction methods should be also discussed. However, it did not seem to have a significant effect on the results of the present study. The key findings were not changed in the subgroup analysis of the subjects who underwent PET/CT with the STE scanner (n = 191, 81.6%; Supplementary Tables 2, 4–7, and Supplementary Figure).

In conclusion, this retrospective analysis of a prospective dataset for a phase II clinical trial shows that tumor FDG uptake on pretreatment PET/CT had a weak correlation with TS expression. However, volume-based tumor FDG uptake, represented as TLG, is independently associated with TS expression status, treatment response, and clinical outcome. These findings suggest that volume-based tumor FDG uptake has a predictive and prognostic value in advanced non-squamous NSCLC. TLG is predictive of treatment response, especially in TS-negative patients treated with gemcitabine/cisplatin. The precise role of tumor FDG uptake should be confirmed in future prospective studies with larger cohorts.

## Supplementary information

Dataset1

## References

[1] Brundage MD, Davies D, Mackillop WJ (2002). Prognostic factors in non-small cell lung cancer: a decade of progress. Chest.

[2] Danenberg PV (1977). Thymidylate synthetase - a target enzyme in cancer chemotherapy. Biochim Biophys Acta.

[3] Wada H, Hitomi S, Teramatsu T (1996). Adjuvant chemotherapy after complete resection in non-small-cell lung cancer. West Japan Study Group for Lung Cancer Surgery. J Clin Oncol.

[4] Sun JM (2015). Pemetrexed Plus Cisplatin Versus Gemcitabine Plus Cisplatin According to Thymidylate Synthase Expression in Nonsquamous Non-Small-Cell Lung Cancer: A Biomarker-Stratified Randomized Phase II Trial. J Clin Oncol.

[5] Scagliotti GV (2008). Phase III study comparing cisplatin plus gemcitabine with cisplatin plus pemetrexed in chemotherapy-naive patients with advanced-stage non-small-cell lung cancer. J Clin Oncol.

[6] Sun JM, Han J, Ahn JS, Park K, Ahn MJ (2011). Significance of thymidylate synthase and thyroid transcription factor 1 expression in patients with nonsquamous non-small cell lung cancer treated with pemetrexed-based chemotherapy. J Thorac Oncol.

[7] Nicolson MC (2013). Thymidylate synthase expression and outcome of patients receiving pemetrexed for advanced nonsquamous non-small-cell lung cancer in a prospective blinded assessment phase II clinical trial. J Thorac Oncol.

[8] Kaira K (2014). (1)(8)F-FDG uptake on PET is a predictive marker of thymidylate synthase expression in patients with thoracic neoplasms. Oncol Rep.

[9] Sasaki R (2005). [18F]fluorodeoxyglucose uptake by positron emission tomography predicts outcome of non-small-cell lung cancer. J Clin Oncol.

[10] Higashi K (2002). 18F-FDG uptake as a biologic prognostic factor for recurrence in patients with surgically resected non-small cell lung cancer. J Nucl Med.

[11] Liu J (2016). Prognostic Value of 18F-FDG PET/CT in Surgical Non-Small Cell Lung Cancer: A Meta-Analysis. PLoS ONE.

[12] Berghmans T (2008). Primary tumor standardized uptake value (SUVmax) measured on fluorodeoxyglucose positron emission tomography (FDG-PET) is of prognostic value for survival in non-small cell lung cancer (NSCLC): a systematic review and meta-analysis (MA) by the European Lung Cancer Working Party for the IASLC Lung Cancer Staging Project. J Thorac Oncol.

[13] Machtay M (2013). Prediction of survival by [18F]fluorodeoxyglucose positron emission tomography in patients with locally advanced non-small-cell lung cancer undergoing definitive chemoradiation therapy: results of the ACRIN 6668/RTOG 0235 trial. J Clin Oncol.

[14] Im HJ (2015). Prognostic value of volumetric parameters of (18)F-FDG PET in non-small-cell lung cancer: a meta-analysis. Eur J Nucl Med Mol Imaging.

[15] de Geus-Oei LF, van der Heijden HF, Corstens FH, Oyen WJ (2007). Predictive and prognostic value of FDG-PET in nonsmall-cell lung cancer: a systematic review. Cancer.

[16] Kaira K (2012). Thymidylate synthase expression is closely associated with outcome in patients with pulmonary adenocarcinoma. Med Oncol.

[17] Kaira K (2010). Biologic correlation of 2-[18F]-fluoro-2-deoxy-D-glucose uptake on positron emission tomography in thymic epithelial tumors. J Clin Oncol.

[18] Atkin GK (2006). The impact of surgically induced ischaemia on protein levels in patients undergoing rectal cancer surgery. Br J Cancer.

[19] Kitagawa Y (2003). FDG-PET for prediction of tumour aggressiveness and response to intra-arterial chemotherapy and radiotherapy in head and neck cancer. Eur J Nucl Med Mol Imaging.

[20] Singh D, Miles K (2012). Multiparametric PET/CT in oncology. Cancer Imaging.

[21] Fathinul F, Nordin AJ, Lau WF (2013). 18[F]FDG-PET/CT is a useful molecular marker in evaluating tumour aggressiveness: a revised understanding of an *in-vivo* FDG-PET imaging that alludes the alteration of cancer biology. Cell Biochem Biophys.

[22] Chattopadhyay S, Moran RG, Goldman ID (2007). Pemetrexed: biochemical and cellular pharmacology, mechanisms, and clinical applications. Mol Cancer Ther.

[23] Mini E, Nobili S, Caciagli B, Landini I, Mazzei T (2006). Cellular pharmacology of gemcitabine. Ann Oncol.

