# Supplementary material for: Predictive and Prognostic Value of 18F-fluorodeoxyglucose Uptake Combined with Thymidylate Synthase Expression in Patients with Advanced Non-Small Cell Lung Cancer
Source: Sci Rep. 2019 Aug 21;9:12215. doi: 10.1038/s41598-019-48674-4 (PMC6704155; doi:10.1038/s41598-019-48674-4)
Supplement: Supplementary file 1 — Dataset1 [file 41598_2019_48674_MOESM1_ESM.pdf]

Predictive and Prognostic Value of ^{18}F-fluorodeoxyglucose Uptake Combined with Thymidylate Synthase Expression in Patients with Advanced Non-Small Cell Lung Cancer

Seung Hwan Moon[1*], Jong-Mu Sun[2*], Jin Seok Ahn[2], Keunchil Park[2], Byung-Tae Kim[1], Kyung-Han Lee[1], Myung-Ju Ahn[2], and Joon Young Choi[1]

[1]Department of Nuclear Medicine, Samsung Medical Center, Sungkyunkwan University School of Medicine, Seoul, Republic of Korea; [2]Division of Hematology-Oncology, Department of Medicine, Samsung Medical Center, Sungkyunkwan University School of Medicine, Seoul, Republic of Korea

[*]These authors contributed equally to this work.

[†]**For correspondence or reprints contact:**

Joon Young Choi, M.D., Ph.D.

Department of Nuclear Medicine, Samsung Medical Center, Sungkyunkwan University School of Medicine, 81 Irwon-ro, Gangnam-gu, 06351 Seoul, Republic of Korea

Tel.: +82-2-3410-2648

Fax: +82-2-3410-2639

E-mail: jynm.choi@samsung.com

Supplementary Table 1. Associations between PET parameters and biomarkers

PET parameters (Continuous value)	TS expression*		*EGFR* mutation†		ALK rearrangement†	
	Correlation coefficient	*P* value	Odds ratio, 95% CI	*P* value	Odds ratio, 95% CI	*P* value
SUV_{max}	0.171	0.009	0.929, 0.871 to 0.991	0.025	1.046, 0.946 to 1.156	0.379
MTV_{Ao}	0.169	0.020	0.996, 0.991 to 0.999	0.043	0.999, 0.992 to 1.006	0.799
TLG_{Ao}	0.191	0.008	0.999, 0.998 to 1.000	0.040	1.000, 0.999 to 1.001	0.816
MTV_{Li}	0.172	0.018	0.996, 0.991 to 1.000	0.049	1.000, 0.995 to 1.007	0.745
TLG_{Li}	0.192	0.008	0.998, 0.998 to 1.000	0.042	1.000, 0.999 to 1.001	0.603
$MTV_{2.5}$	0.142	0.040	0.994, 0.989 to 0.999	0.039	1.001, 0.995 to 1.008	0.738
$TLG_{2.5}$	0.185	0.011	0.999, 0.998 to 1.000	0.041	1.000, 0.999 to 1.001	0.583

TS, thymidylate synthase; *, Spearman's rank test; *MTV*, metabolic tumor volume; *TLG*, total lesion glycolysis; *SUV*, standard uptake value; $_{Ao}$, aortic arch; $_{Li}$, liver; $_{2.5}$, fixed value of SUV 2.5; *EGFR*, epidermal growth factor receptor; *ALK*, anaplastic lymphoma kinase; †, logistic regression analysis.

Supplementary Table 2. Associations between PET parameters and biomarkers (Subgroup analysis with STE scanner group; n = 191)

PET parameters (Continuous value)	TS expression*		*EGFR* mutation†		ALK rearrangement†	
	Correlation coefficient	*P* value	Odds ratio, 95% CI	*P* value	Odds ratio, 95% CI	*P* value
SUV_{max}	0.195	0.007	0.919, 0.854 to 0.989	0.024	1.078, 0.972 to 1.195	0.156
MTV_{Ao}	0.201	0.012	0.992, 0.985 to 0.999	0.017	1.000, 0.994 to 1.007	0.924
TLG_{Ao}	0.239	0.003	0.998, 0.997 to 1.000	0.017	1.000, 0.999 to 1.001	0.562
MTV_{Li}	0.201	0.012	0.991, 0.983 to 0.998	0.017	1.001, 0.994 to 1.007	0.843
TLG_{Li}	0.239	0.003	0.998, 0.997 to 1.000	0.017	1.000, 0.999 to 1.001	0.521
$MTV_{2.5}$	0.188	0.019	0.989, 0.980 to 0.998	0.018	1.001, 0.994 to 1.008	0.800
$TLG_{2.5}$	0.236	0.003	0.998, 0.997 to 1.000	0.018	1.000, 0.999 to 1.001	0.499

TS, thymidylate synthase; *, Spearman's rank test; *MTV*, metabolic tumor volume; *TLG*, total lesion glycolysis; *SUV*, standard uptake value; $_{Ao}$, aortic arch; $_{Li}$, liver; $_{2.5}$, fixed value of SUV 2.5; *EGFR*, epidermal growth factor receptor; *ALK*, anaplastic lymphoma kinase; †, logistic regression analysis.

Supplementary Table 3. Results of biomarkers according to TS expression

	TS expression		*P* value*
	Positive (N = 115)	Negative (N = 119)	
High TLG (> 88)	70 (60.8%)	49 (41.1%)	0.007
EGFR mutation	28 (24.3%)	49 (41.2%)	0.014
ALK rearrangement	4 (3.5%)	10 (8.4%)	0.167†

TS, thymidylate synthase; *, Chi-square test; *SUV*, standard uptake value; *EGFR*, epidermal growth factor receptor; *ALK*, anaplastic lymphoma kinase; †, Fisher's exact.

Supplementary Table 4. Biomarkers according to TS expression (Subgroup analysis with STE scanner group; n = 191)

	TS expression		*P* value*
	Positive (N = 96)	Negative (N = 95)	
High TLG (> 88)	51 (53.1%)	30 (31.6%)	0.003
EGFR mutation	23 (23.9%)	38 (40.0%)	0.017
ALK rearrangement	3 (3.1%)	8 (8.4%)	0.213†

TS, thymidylate synthase; *, Chi-square test; *SUV*, standard uptake value; *EGFR*, epidermal growth factor receptor; *ALK*, anaplastic lymphoma kinase; †, Fisher's exact.

Association of high TLG with TS positivity: Odds ratio = 2.64, 95% CI = 1.38 to 5.04, P = 0.003 (Logistic regression analysis).

Supplementary Table 5. Factors associated with objective response rate (Subgroup analysis with STE scanner group; n = 191)

	Univariate			Multivariate		
	OR	95% CI	*P*-value	OR	95% CI	*P*-value
AP group (vs. GP group)	2.29	1.26-4.16	0.006	3.11	1.53-6.32	0.002
High TLG (vs. low TLG)	1.99	1.02-3.86	0.042	2.31	1.12-4.73	0.023
Male (vs. female)	0.57	0.32-1.03	0.064	0.38	0.19-0.78	0.008
EGFR wild-type (vs. mutation)	1.50	0.79-2.84	0.210			
TS-positive (vs. TS-negative)	1.12	0.63-2.00	0.833			
Ever smoked (vs. never smoker)	0.95	0.53-1.69	0.853			
Age ≥ 65 years (vs. < 65 years)	0.87	0.47-1.63	0.670			
ALK rearrangement	1.33	0.39-4.52	0.649			

OR, odds ratio; CI, confidence interval; AP, pemetrexed/cisplatin-treated; GP, gemcitabine/cisplatin-treated; EGFR, epidermal growth factor receptor; TS, thymidylate synthase; ALK, anaplastic lymphoma kinase

Supplementary Table 6. Factors associated with poor overall survival (Subgroup analysis with STE scanner group; n = 191)

	Univariate			Multivariate*		
	HR	95% CI	*P*-value	HR	95% CI	*P*-value
Male (vs. female)	2.97	1.82-4.86	<0.001	2.29	1.29-4.05	0.005
EGFR wild-type (vs. mutation)	3.53	1.91-6.52	<0.001	2.60	1.30-5.19	0.007
TS-positive (vs. TS-negative)	2.24	1.43-3.52	<0.001	1.84	1.07-3.18	0.028
Age ≥ 65 years (vs. < 65 years)	2.28	1.47-3.53	<0.001	1.99	1.19-3.33	0.009
High TLG	2.40	1.41-4.09	0.012	1.74	1.01-3.01	0.046
Ever smoked (vs. never smoker)	2.90	1.77-4.77	<0.001			
AP group (vs. GP group)	1.03	0.67-1.58	0.900			
ALK rearrangement	0.92	0.34-2.52	0.871			

HR, hazard ratio; CI, confidence interval; EGFR, epidermal growth factor receptor; TS, thymidylate synthase; TLG, total lesion glycolysis; AP, pemetrexed/cisplatin-treated; GP, gemcitabine/cisplatin-treated; ALK, anaplastic lymphoma kinase; * Forward stepwise method

Supplementary Table 7. Factors associated with poor progression-free survival (Subgroup analysis with STE scanner group; n = 191)

	Univariate			Multivariate*		
	HR	95% CI	*P*-value	HR	95% CI	*P*-value
Male (vs. female)	1.62	1.19-2.21	0.002	1.61	1.15-2.24	0.005
AP group (vs. GP group)	0.71	0.52-0.96	0.028	0.65	0.46-0.91	0.013
High TLG	1.50	1.07-2.10	0.019	1.49	1.06-2.09	0.020
Ever smoked (vs. never smoker)	1.42	1.04-1.92	0.025			
Age ≥ 65 years (vs. < 65 years)	1.42	1.03-1.96	0.030			
TS-positive (vs. TS-negative)	1.40	1.03-1.91	0.032			
EGFR wild-type (vs. mutation)	1.14	0.83-1.57	0.428			
ALK rearrangement	0.89	0.47-1.68	0.715			

HR, hazard ratio; CI, confidence interval; TLG, total lesion glycolysis; TS, thymidylate synthase; AP, pemetrexed/cisplatin-treated; GP, gemcitabine/cisplatin-treated; EGFR, epidermal growth factor receptor; ALK, anaplastic lymphoma kinase; * Forward stepwise method

Supplementary Figure. Response rates according to TLG and treatment arm (Subgroup analysis with STE scanner group; n = 191)

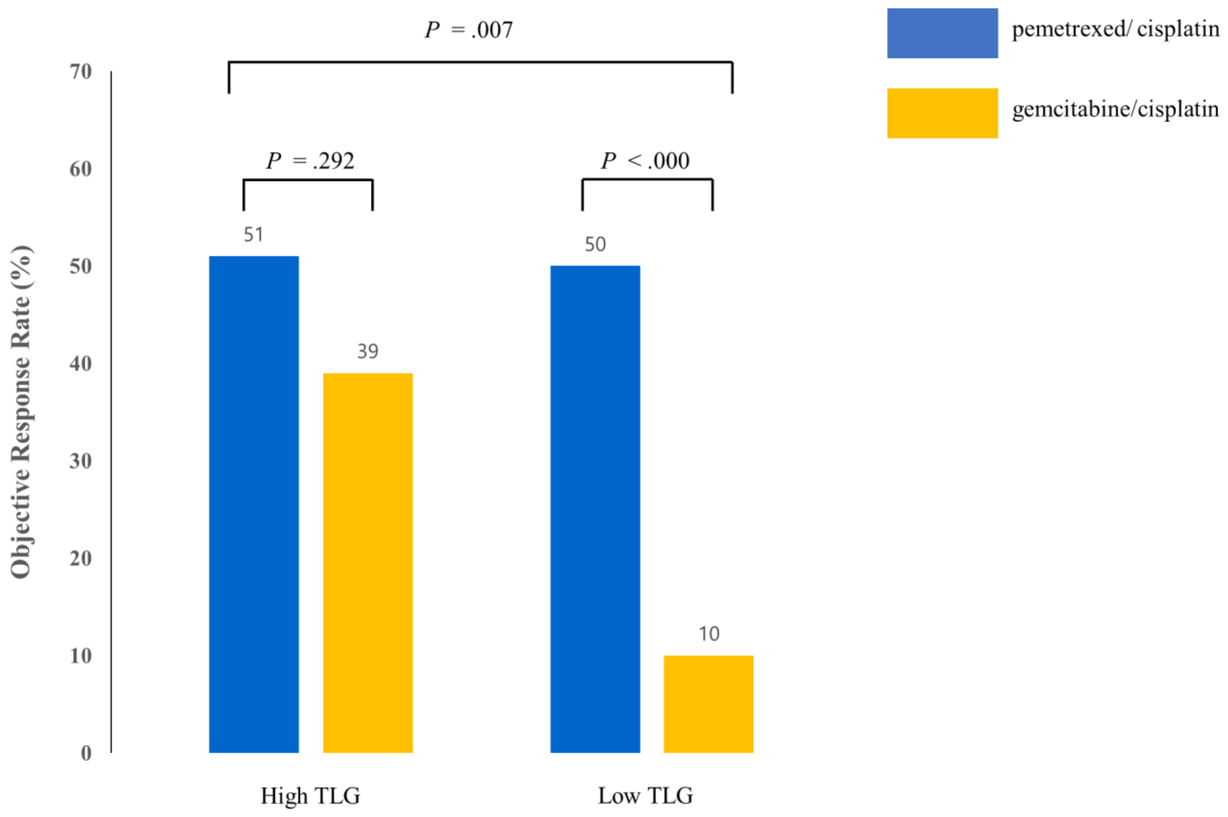

The response rate for the pemetrexed/cisplatin arm was significantly higher than that for the gemcitabine/cisplatin arm in the low TLG group, whereas response rates were not significantly different between the treatment arms in the high TLG group.
